# Acute pancreatitis secondary to hypercalcemia in a patient with primary hyperparathyroidism: An uncommon association

**DOI:** 10.1016/j.amsu.2022.104832

**Published:** 2022-11-05

**Authors:** Inass Arhoun EL Haddad, Amine Elmouhib, Safaa Kachmar, Sanae Elmezzioui, Adelmounain Daoudi, Imane Melhaoui, Amal Mojahid, Houssam Bkiyar, Imane kAMAOUI, Brahim Housni

**Affiliations:** a Department of Intensive Care Unit, Mohammed VI University Hospital, Oujda, Morocco; b Radiology Department, Mohammed VI University Hospital, Faculty of Medicine and Pharmacy, Oujda, Morocco; c Nephrology Department, Mohammed VI University Hospital, Faculty of Medicine and Pharmacy, Oujda, Morocco; d Faculty of Medicine and Pharmacy, Mohammed 1 st University, Oujda, Morocco; e Mohammed First University Oujda, FMP Oujda, LAMCESM, Oujda, Morocco

**Keywords:** Acute pancreatitis, Hypercalcemia, Primary hyperparathyroidism

## Abstract

**Introduction:**

An uncommon cause of acute pancreatitis, primary hyperparathyroidism accounts for less than 1% of cases.

**Case presentation:**

A 41-year-old male with acute pancreatitis and hypercalcemia is described in this case. Primary hyperparathyroidism was discovered during the work-up for hypercalcemia. During the first 24 hours after his hospitalization, the patient was monitored in the intensive care unit, and after a positive outcome, he was discharged.

**Discussion:**

Pancreatitis is a rare presentation of hyperparathyroidism. The first documented case of this association was by Erdheim in 1903 on a post-mortem study (2). Hyperparathyroidism is often only discovered after two or three episodes of recurrent pancreatitis (5), thankfully, in this case, the patient has been diagnosed from its first episode and eventually treated to prevent any other ones. hypercalcaemia leads to increase calcium in the pancreatic responsible for aggression of the pancreatic parenchyma and ducts, Other authors suggest that the pancreatic secretion in patients with hypercalcaemia is lower than normal, but the enzyme activity remains normal, resulting in the formation of protein plugs in the pancreatic ducts leading to their obstruction and self-digestion.

**Conclusion:**

Hypercalcemia can cause acute pancreatitis. This report describes rare case of a patient with acute pancreatitis caused by hyperparathyroidism.

## Highlights

• Primary Hyper Parathyroidism is rarely complicated with acute pancreatitis, making it a very interesting revealing symptom.
• The causality of HPT is only incriminated in the pancreatitis after recurrent episodes or while progressing to the chronic versus, in our case we diagnosed it form it's very first onset.
• Its management required the implication of different disciplines such as the ICU, Radiology, endocrinology, gastrology but also nephrology.

## 1 Introduction

The association between pancreatitis and hyperparathyroidism (HPT) has been addressed in the literature since 1957, when Cope et al. first brought attention to it [1]**.** HPT is currently thought to be a cause of acute and chronic pancreatitis. However, no solid explanation of the pathophysiological processes is provided, which is why some writers question the link. Nevertheless, it is apparent that the hypercalcaemia associated with HPT plays a significant role in the pathogenesis of pancreatitis and is the primary key criteria in the diagnosis of HPT. We present a case of a severe acute pancreatitis with primary HPT in a patient with no additional risk factors for pancreatitis; we explain the key pathophysiological processes involved in this relationship.

## 2 Presentation of the clinical case

We report the case of a 41 years old male patient**with no prior co morbidities**, presenting to the emergency department for an intense stomachache evolving for 3 days, the other signs included nausea and fever.

At admission, the patient was conscious, and stable on both hemodynamic and respiratory levels (BP at 120/71 mmHg, heart rate at 95/min, respiratory rate at 18/min, and the oxygen pulsated saturation at 99%), the abdominal examination showed a diffuse abdominal sensitivity, a fever at 39.7 °C; the glycemia level was high at 3.41g/l, with a 2-cross glycosuria on urine dipstick but with no acetonuria.

Biologically, the lipasemia level was high at 2010 which represents more than 3 times the normal rate, so the diagnosis of an acute pancreatitis was confirmed, the inflammatory assessment reveled a hyperleucocytose at 13 777/mm3 with a predominance of the PNN at 10 450/mm3, the C-reactive protein level was at 253g/l, the patient had also an acute kidney injury(AKI) with a Creatinine at 30 g/l and an urea level at 0.78g/l, since it had no influence on the dieresis, the AKI was classified as KDIGO 2. The blood ionogram revealed a hyper calcemia at 3.7 mmol/l (the ionized calcemia was not meseared due to a technical laboratory problem), and the parathormon level was as elevated at 809 g/dl with a hypo phosphoremia at 0.2 mmol/l.

To evaluate the cardiac impact of the hyper calcemia, we have realized an electrocardiogram that showed no signs of influence notably no decreased QT space and no arrhythmia.

Radiological exploration included an abdominal CT scan revealing a pancreas increased in size, losing its physiological lobulations, heterogeneously enlarged after injection of contrast, containing some hypodense areas of necrosis, associated with significant infiltration of the surrounding fat with multiple peri-pancreatic necrosis flows, opposite the anterior para-renal fascia, bilaterally peri-splenic at the level of the parietal colic gullet and fusing at the mesenteric level, thus the pancreatitis was classified as severe (CSTI at 8 following the BALTHAZAR classification), to be noted that the splenic vein has a thrombus (Fig. 1a, Fig. 1ba and b).

**Fig. 1a:**
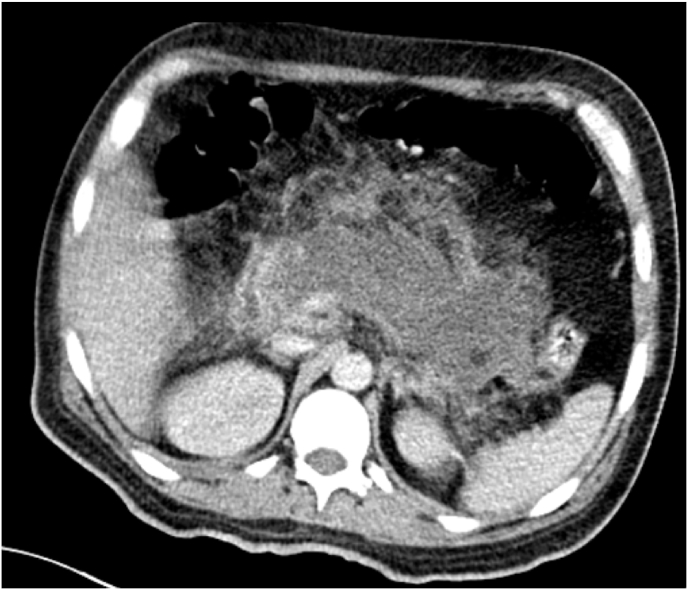
abdominal CT scan revealing a pancreas increased in size, losing its physiological lobulations, heterogeneously enlarged after injection of contrast, containing some hypodense areas of necrosis, associated with significant infiltration of the surrounding fat with multiple peri-pancreatic necrosis flows, opposite the anterior para-renal fascia, bilaterally peri-splenic at the level of the parietal colic gullet and fusing at the mesenteric level.

**Fig. 1b:**
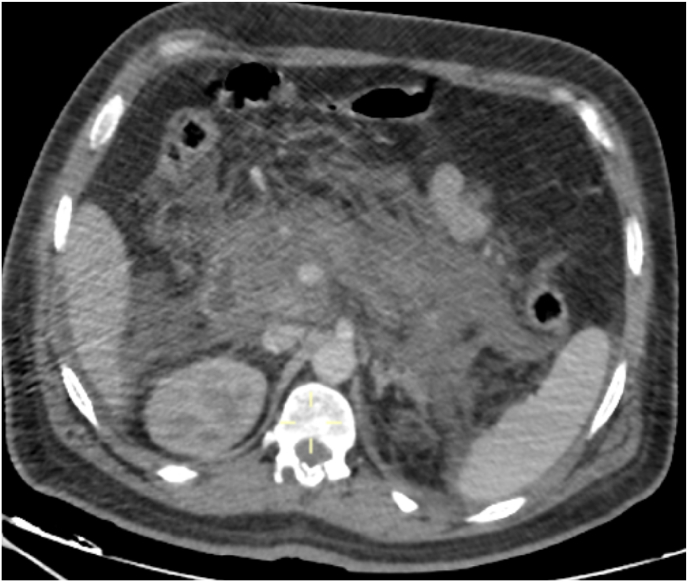
abdominal CT scan revealing a pancreas increased in size, losing its physiological lobulations, heterogeneously enlarged after injection of contrast, containing some hypodense areas of necrosis, associated with significant infiltration of the surrounding fat with multiple peri-pancreatic necrosis flows, opposite the anterior para-renal fascia, bilaterally peri-splenic at the level of the parietal colic gullet and fusing at the mesenteric level.

He was diagnosed with acute pancreatitis secondary to hypercalcemia, since hypercalcemia is known to trigger acute pancreatitis.

The patient have been admitted to the intensive care unit for a 24h observation, there he had a treatment that consisted of:

• symptomatic treatment for vomiting and abdominal pain
• Rehydration with SS 0.9% at a rate of 4–6 l/day.
• Inhibition of bone resorption by administration of zoledronic acid at a dose of 4 mg.
• Anticoagulation with INNOHEP (UFH) at the dose of 14 000 UI/day.
• Insulin to regulated the glycemia level.

After 3 days in ICU, the control assessment showed a decrease in the calcemia level going from 3.7 to 2.1mmol/l and that of creatinine going from 30 to 15, there was no abdominal pain, and the patient afterwards was transferred to endocrinology department for further exploration and treatment.

**Fig. 2:**
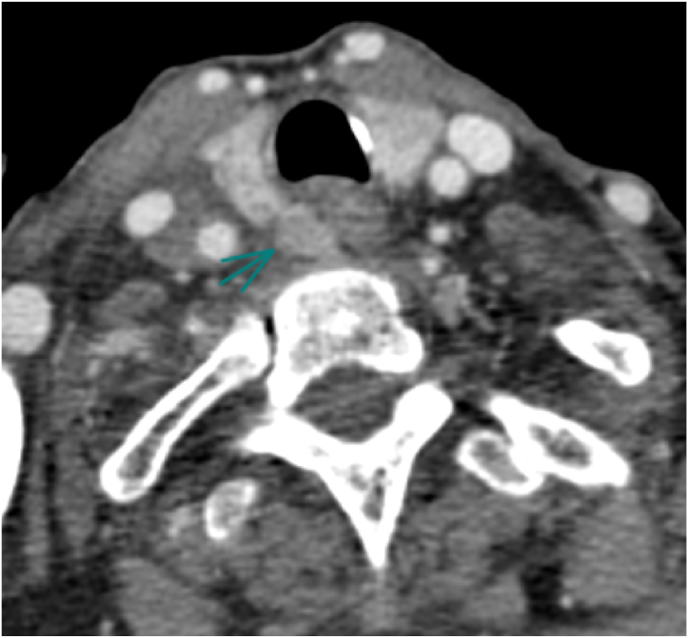
cervical CT scan reveling the adenoma with no surgical indication.

The patient was hospitalized at our department, the ICU, for 24 hours for surveillance as the pancreatitis was classified severe, but soon after he was transferred to the endocrinology department, where the exploration of the hyperparathyroidism were continued, the patient benefited from a cervical echocardiography in search of adenoma, but it was not found, therefore a cervical CT scan was performed reveling the adenoma, it had no surgical indication(Fig. 2).

2 months later, to follow the patient's progress, another abdominal scan was performed, revealing that the pancreas necrosis evolved to false cyst of the pancreas (Fig. 3a, Fig. 3ba,b), so the patient is monitored regularly thought out consultation with the endocrinology department till this day.

**Fig. 3a:**
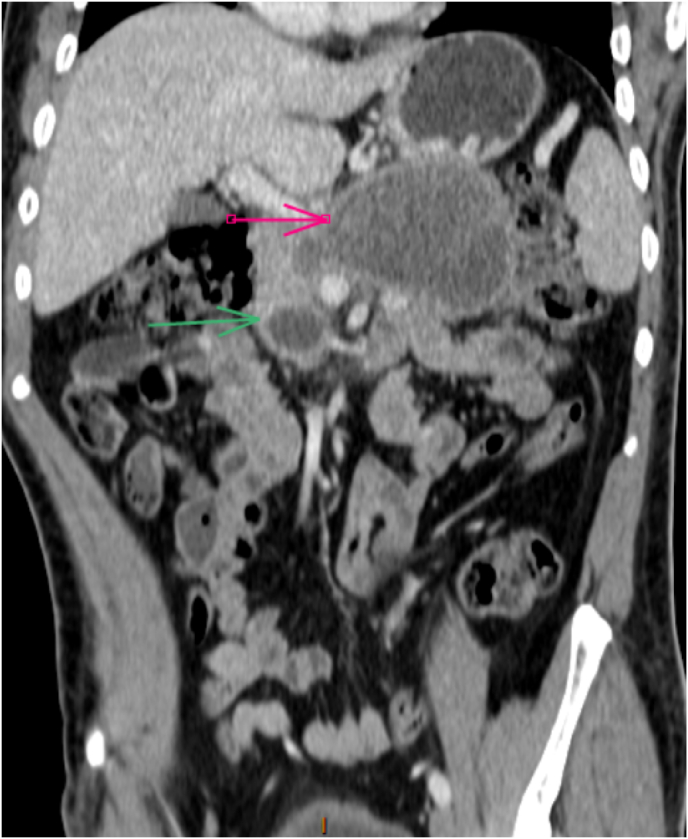
abdominal scan revealing that the pancreas necrosis evolved to false cyst of the pancreas.

**Fig. 3b:**
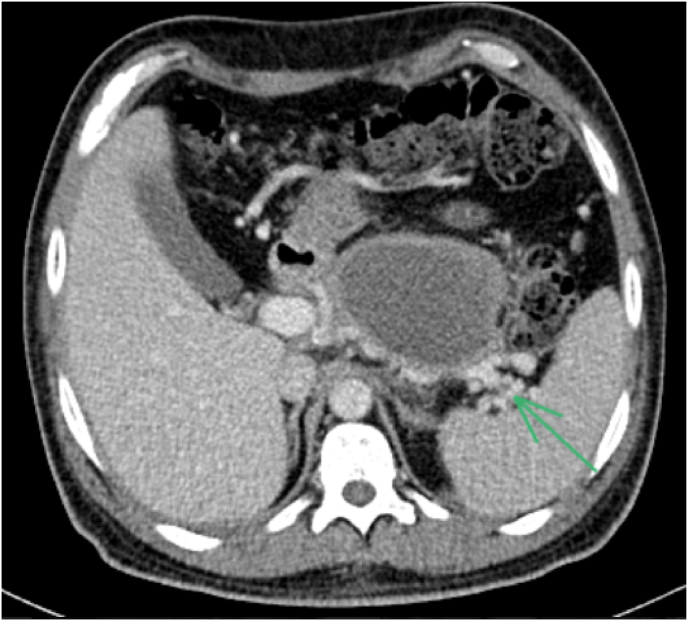
abdominal scan revealing that the pancreas necrosis evolved to false cyst of the pancreas.

This case is written following the SCARE guidelines [14].

## 3 Discussion

Pancreatitis is a rare presentation of hyperparathyroidism. The first documented case of this association was by Erdheim in 1903 on a post-mortem study [2]**.** It was not until 1957, for Cope et al. to draw attention to this association by publishing the publication of two cases [1]**.** The incidence of pancreatitis linked with hyperparathyroidism has been found to range from 1.5% to 15.3% in individuals aged 20 to 70 [3]**.** The prevalence is decreasing, and more recent research show that it is approximately 1% [4]**.**

Hyperparathyroidism is often only discovered after two or three episodes of recurrent pancreatitis [5]**.** thankfully, in this case, the patient has been diagnosed from its first episode and eventually treated to prevent any other ones.

Kelly et al. have shown that hypercalcaemia leads to increase in calcium in the pancreatic juice and accelerate conversion of trypsinogen to trypsin responsible for aggression of the pancreatic parenchyma and ducts [2]**.** The occurrence of pancreatitis in other states of major hypercalcaemic conditions such as bone metastases, parenteral nutrition, and vitamin D intoxication confirms this hypothesis. Other authors suggest that the pancreatic secretion in patients with hypercalcaemia is lower than normal, but the enzyme activity remains normal, resulting in the formation of protein plugs in the pancreatic ducts leading to their obstruction and self-digestion of the pancreas [6]**.** Pancreatic duct obstruction could also be due to calcifications [7]**.** Parathyroid hormone is thought to play a role in the pathogenesis of pancreatitis by inhibiting pancreatic vascularisation or by causing the formation of micro thrombi leading to necrosis of the pancreatic parenchyma [4]**.**

The acute onset of persistent and severe epigastric pain, often radiating to the back, is a typical symptom of acute pancreatitis. Patients with acute pancreatitis caused by hyperparathyroidism also complain of abdominal pain [8], and so did our patient. He fulfilled the diagnostic criteria for Systemic Inflammatory Response Syndrome [9]**.**, including heart rate >90/min, a fever at 39.7 °C, and white blood cell count >12 000/mm3. Although sepsis was suspected, septic shock did not occur.

CT and MRI imaging have their greatest implication on disease severity >72 hours after onset of symptoms. Such imaging is helpful in classifying severity of disease, correlating mortality and treatment plans. Our patient presented to the ER after 3 days of ongoing pain allowing us to perform a ST scan without any delay, and to classify the AP as severe following the Balthazar classification.

As of the initial management of acute pancreatitis secondary to primary hyperparathyroidism hypercalcemia [10,11]**.**, it depends on this main therapeutic strands:

• Fluid resuscitation: In those patients with severe hypercalcemia and minimal comorbidities, 4–6 L can be administered over the first 24 h. In this case, normal saline may be a preferred option, as lactated ringers contain calcium. Just after normovolemia is established, either oral or maintenance i.v. fluids is continued to maintain satisfactory urine output (0.5–1 ml/kg/h) until the anti-hypercalcemic agents start giving effect [12]**.**
• Enteral nutrition: It is thought that enteral nutrition maintains gut integrity, thereby limiting translocation of bacteria and a resultant inflammatory cascade. Early initiation of enteral nutrition, within 48 hours of presentation, may improve mortality and lower the likelihood of needing surgery, it can be administered orally in cases of mild and moderate AP or through a nasogastric (NG) tube to ensure a caloric target of 35–40 kcal/kg/d [13]**.**
• Medication [11]:
  oDiuretic treatment with loop diuretic (calciuric action) but only after rehydration and with diuresis compensation.
  oInhibition of bone resorption using 2 treatments:
    ⁃ Intravenous biphosphonate: reference treatment especially if hypercalcaemia of neoplastic origin.
    ⁃ Calcitonin 4IU/kg/6h by subcutaneous injection (that was not used due to a rupture in the hospital).
  o Preventive anticoagulation: In our case the patient had a splenic thrombosis which explain the curative dose administrated, as for the choice of the molecule, he had an acute kidney injury, so we opted for an unfractionned heparin UHF.
  o Antibiotic therapy: no preventive antibiotic therapy is the rule in acute pancreatitis; it should be administrated only in case of a surinfection of the necrosis areas and after performing a puncture with drainage of the collections.
• Endoscopic management [12]: in case a calcification or an obstruction of the biliary duct has been found. The role of ERCP in AP is to relieve ampulla obstruction that may result in persistent AP or cholangitis.
• Hemodialysis [11] to be discussed in case of severe hypercalcaemia and especially in case of renal failure.

## 4 Conclusion

Hypercalcemia can cause acute pancreatitis. This report describes rare case of a patient with acute pancreatitis caused by hyperparathyroidism.

## Provenance and peer review

Not commissioned, externally peer reviewed.

## Ethical approval

This is a case report that does not require a formal ethical committee approval. Data were anonymously registered in our database. Access to data was approved by the head of the department.

## Annals of Medicine and Surgery

The following information is required for submission. Please note that failure to respond to these questions/statements will mean your submission will be returned. If you have nothing to declare in any of these categories then this should be stated.

## Sources of funding

This research was not funded

## Author contribution

Dr Inass Arhoun El Haddad and Dr. Amine Elmouhib: are principal investigators that collected and analyzed data, wrote the manuscript and prepared the final draft for the submission. Prof. Brahim Housni and Prof. Hanane Latraache: supervised the research project and approved the final draft for publication All authors approved the final version of the manuscript.

## Registration of research studies

This is not an interventional study. We only reported the patients’ findings from our database as a case series.

## Guarantor

Dr Inass Arhoun El Haddad & Dr Amine Elmouhib.

## Consent

Written informed consent was obtained from the patient for publication of this case report and accompanying images. A copy of the written consent is available for review by the Editor-in-Chief of this journal on request.

## Declaration of competing interest

The authors declare no conflict of interest.
